# Two New Therapies in the Cocaine-dependents: Comparison of Topiramate and Contingency Management

**Published:** 2018-10

**Authors:** Bijan PIRNIA, Ali Akbar SOLEIMANI, Abbas TAVALLAII, Rasool ROSHAN, Kambiz PIRNIA

**Affiliations:** 1. Dept. of Psychology, Faculty of Humanities, University of Science and Culture, Tehran, Iran; 2. Dept. of Psychiatry, Faculty of Medicine, Behavioral Research Center, Baqyiatallah University of Medical Sciences, Tehran, Iran; 3. Dept. of Psychology, Faculty of Humanities, Shahed University, Tehran, Iran; 4. Bijan Center for Substance Abuse Treatment, Tehran, Iran

## Dear Editor-in-Chief

Topiramate (TPM) is a kind of anticonvulsant drug that in 1996 was suggested by Food and Drug Administration (FDA) as curing drug of adults’ convulsion. TPM facilitates gamma amino butyric acid (GABA) transference and prevents Glutamates transference, where this process can reduce the cocaine reinforcing effect and can be used in management of cocaine craving (1). There is an assumption that convulsion can be accounted as a running mechanism in addiction. Therefore, anticonvulsant drugs are counted as suitable candidates for intervention in addiction domain of cocaine craving (2).

One of the modern therapies in the realm of drug abuse is contingency management (CM). Contingency management shapes behavior in form of using secondary positive boosters such as coupons, goods, and services. Contingency management about wide range of drugs (stimulants, alcohol, marijuana, tobacco) has been approved (3), although the research findings of efficacy of TPM and contingency management therapy are contradictive. Therefore, this study aimed to examine the effectiveness of the TPM and contingency management compared to the placebo.

The study was a second part of a process of research in the form of a placebo-controlled in Bijan Center for Substance Abuse Treatment in Tehran, after achieving a period of cocaine abstinence from 15 Sep to 15 Nov 2014, 100 patients (Age range=18–35; SD=2.25). Cocaine-dependents were randomly selected and were assigned to four groups (n=25) of TPM, Contingency Management, Mix and the placebo control group. In TPM group, participants were received escalating doses. In addition, contingency management was provided for twelve weeks. All subjects received brief behavioral compliance enhancement treatment (BBCET). The urine test with assumed threshold of 300 ng/ml. Primary outcome measures included twelve weekly urine drug screens (detection of benzoylecgonine). Secondary outcome measures included cocaine craving in pretest and posttest. Generalized estimating equations (GEE) models analyzed the data.

The mean of (95% of confidence) number of negative cocaine urine tests was 13.09 (9.48–16.70) in Contingency Management group, 12.91 (7.26–13.84) in mix group and 11.83 (6.65–17.01) in control group (*P*>0.05). But Contingency Management has significant effect on reducing the craving in CM and mix group (Table 1). Moreover, TPM was not better than placebo in reducing cocaine use. However, in secondary outcome, TPM was better than placebo in reducing cocaine craving, Moreover, the highest variance explaining the changes in craving was assigned to the combined treatment (*P*<0.01).

**Table 1:** Multivariate GEE analysis of predictors for ongoing drug use during 3 month TPM in urine cocaine test + cocaine craving

***Variable***	***Topiramate N=25***	***CM N=25***	***MIX N=25***	***Placebo N=25***	***Pairwise Comparisons***
Urine	12.24 (1.75)	13.09 (1.4)	12.91 (2.19)	11.83 (2.01)	NS
Cocaine Test					
Cocaine craving	15.04 (2.73)	16.11 (3.74)	14.21 (3.07)	17.52 (3.82)	*P*<0.01

All three types of treatment played a significant efficacy in reducing the craving. Moreover, the highest variance explaining the changes in craving was assigned to the combined treatment. Furthermore, TPM was not better than placebo in reducing cocaine use. The findings are inconsistent with the findings of other studies. Prescription of 300 milligram TPM per day was reported effective in remedy of cocaine reliance (4).

The present study is different in two components with other research. The first one is the sample size and second is the basic dose of 50 mg was used but in the present study, the basic dose of 25 mg was used. Moreover, TPM has reduced the weekly mean of urine test of methamphetamine between 6 to 12 wk (5). Inconsistent with our findings, the effectiveness of TPM was reported on increasing negative urine test of methamphetamine in a sample of Iranian users in the sixth week, significant (6). In Consistent with the findings of this study, CM is associated with increased rates of negative urine test in cocaine users (7).

Future studies could focus on enhancing the effectiveness of CM and at the same time on considering to the savings aspect in using of boosters and examining the possible side effects of topiramate.
